# Survival analysis of 230 patients with unresectable hepatocellular carcinoma treated with bland transarterial embolization

**DOI:** 10.1371/journal.pone.0227711

**Published:** 2020-01-14

**Authors:** Ezio Lanza, Riccardo Muglia, Isabella Bolengo, Dario Poretti, Felice D’Antuono, Roberto Ceriani, Guido Torzilli, Vittorio Pedicini

**Affiliations:** 1 Department of Diagnostic and Interventional Radiology, Humanitas Clinical and Research Hospital IRCCS, Rozzano, Milan, Italy; 2 Training School in Radiology, Humanitas University, Pieve Emanuele, Milan, Italy; 3 Department of Internal Medicine - Hepatology, Humanitas Clinical and Research Hospital IRCCS, Rozzano, Milan, Italy; 4 Department of Surgery - Hepatobiliary and General Surgery, Humanitas Clinical and Research Hospital IRCCS, Rozzano, Milan, Italy; Texas A&M University, UNITED STATES

## Abstract

Locoregional therapies for hepatocellular carcinoma (HCC) include endovascular treatments such as chemoembolization (TACE) and bland embolization (TAE). TACE is the most adopted technique, despite a lack of definitive evidence of superiority over TAE, which is less costly and better tolerated due to the absence of chemotherapy. However, few studies have reported data on TAE monotherapy for unresectable HCC. We report our results in a cohort of 230 patients with unresectable HCC treated with TAE (TAE with 40-100micron microparticles, TAE with microparticles plus n-butyl-2-cyanoacrylate, TAE with Lipiodol) over the course of seven years. Thirty-seven patients (14%) were down-staged during observation and also received a percutaneous ablation. We observed 1-, 2-, 3-, 4- and 5-year rates of 84,8%, 58,7%, 38,3%, 28,3%, and 18,7%. Patients who also received percutaneous treatment performed best. Our results broaden the body of evidence for the use of TAE in advanced HCC.

## Introduction

Transarterial chemoembolization (TACE) is probably the most studied locoregional therapy for unresectable hepatocellular carcinoma (HCC) and is included in treatment algorithms of the European Association for the Study of the Liver (EASL), the Barcelona Clinic Liver Cancer (BCLC) and the American Association for the Study of Liver Disease (AASLD) [1].

The term TACE is strictly related to the use of chemotherapeutic drugs in the compound injected intra-arterially; however, the advantage provided by chemotherapy compared to bland transarterial embolization (TAE) is unproven, whereas more adverse events and higher costs are reported [2,3]. This is due to the intrinsic chemoresistance of HCC [4], and TACE has not shown higher survival rates compared to TAE, mainly when this latter is performed with small microparticles [2,5]. Nonetheless, studies reporting survival data of HCC patients treated with TAE alone, such as the present paper, are few [6].

In this manuscript we report our experience in performing TAE in patients with advanced HCC, aiming to compare our results to heterogeneous literature about TAE and to broaden the body of evidence in favor of performing TAE instead of TACE.

## Materials and methods

The Institutional Review Board of IRCCS Istituto Clinico Humanitas (Rozzano, Italy) approved this retrospective, observational review of HCC TAEs performed between January 1st, 2011 and February 28th, 2018 in a single center.

All data for the purposes of this study was retrieved from the Institution digital archive, was fully anonymized and analyzed between June 2018 and October 2018. All patients provided informed written consent to have their data records used in research.

The indications to treat were given by a multidisciplinary team comprising hepatologists, interventional radiologists, liver surgeons, oncologists, nuclear medicine physicians, and radiotherapists [7], who discussed each case.

The inclusion criteria for this study were: A) histological or imaging diagnosis of HCC, in accordance to the EASL clinical practice guidelines [8]; B) unresectable HCCs treated with one or more TAE. Patients who also received percutaneous treatments (PT) such as thermal ablation (intended as microwave [MW] or radiofrequency [RF]) or percutaneous ethanol injection (PEI) were admitted. PT was always suggested as a result of a multidisciplinary team discussion, which confirmed a temporary BCLC down-staging secondary to complete response to the embolization.

**Fig 1:**
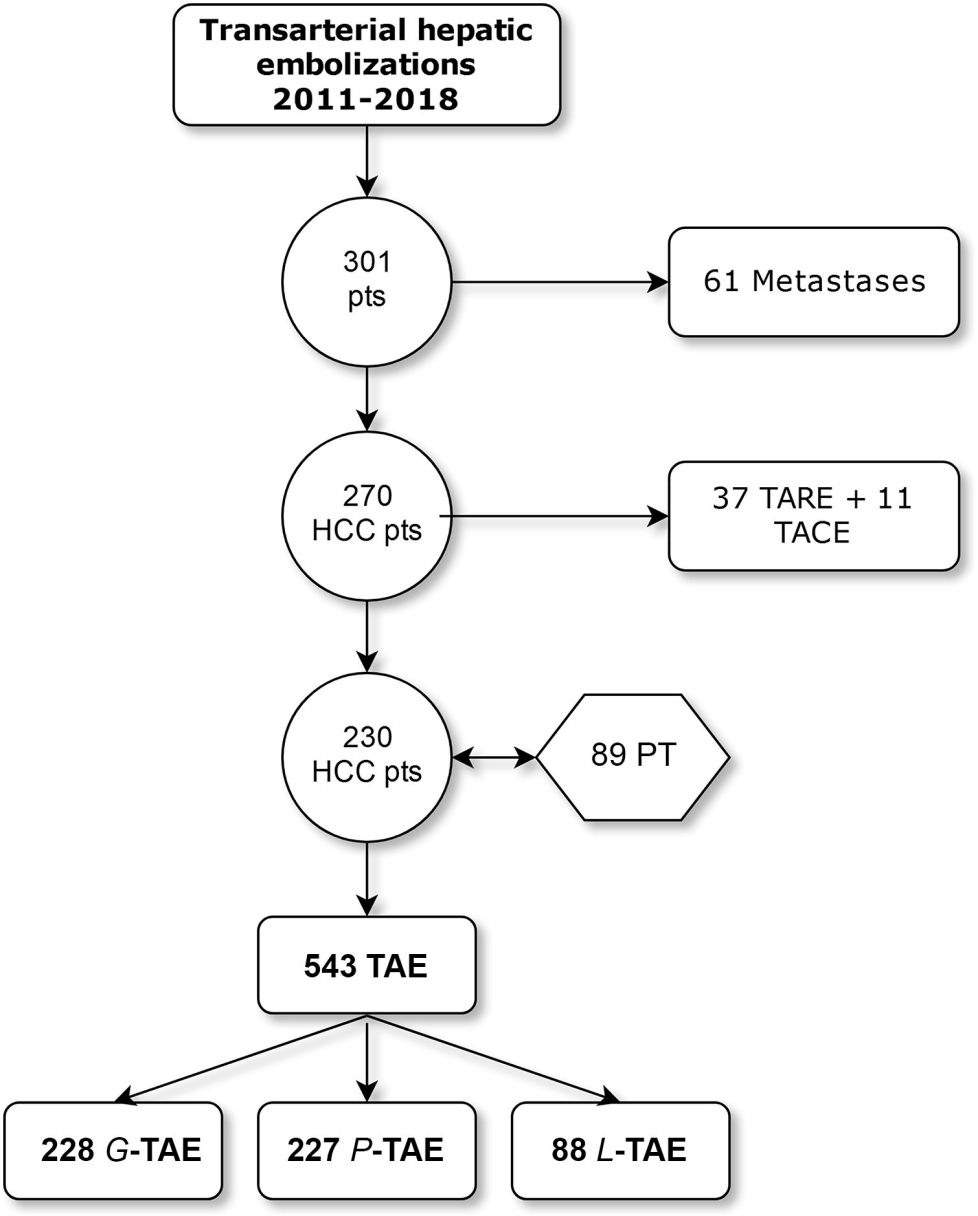
Flow-chart of the inclusion process.

Patients who received TACE or radioembolization (TARE) during the observation period were excluded. The inclusion process is shown in Fig 1.

We analyzed the following characteristics for every patient: number and technique of all endovascular therapies received, cirrhotic subset (HCV, HBV, alcohol), previous/concurrent locoregional treatments received, concurrent or successive neoplasm other than HCC, the Child-Pugh score, BCLC score, duration of embolization (retrieved from digital records) and survival time from the first endovascular treatment. For patients lost to follow-up, we considered the last available check-up at our hospital to estimate survival. The end date of the observation period was set to May 1st, 2018.

All procedures were performed by two senior operators each with more than ten years’ experience (PD and PV) and two junior interventional radiologists (TM and LE) with at least three years’ experience, in an angiographic suite dedicated to interventional radiology (V5000 Philips Medical System, Amsterdam, The Netherlands), equipped with cone-beam CT (Siemens Artis-Zee, Munich, Germany) from 2014.

HCC response to TAE was evaluated with CT or MRI after one month, followed by a clinical examination and multidisciplinary discussion. Whenever any viable tumor could be observed (complete response), the patient was kept under trimestral radiological and clinical control [9]; for partial response or stable disease, another TAE could be performed; in case tumor progression was encountered at the first control, a medical therapy could be proposed, according to international guidelines [10]. Nonetheless, a patient-tailored approach based on the multidisciplinary evaluation was endorsed for each case.

### TAE technical variations

#### Microparticles TAE (P-TAE)

**Fig 2:**
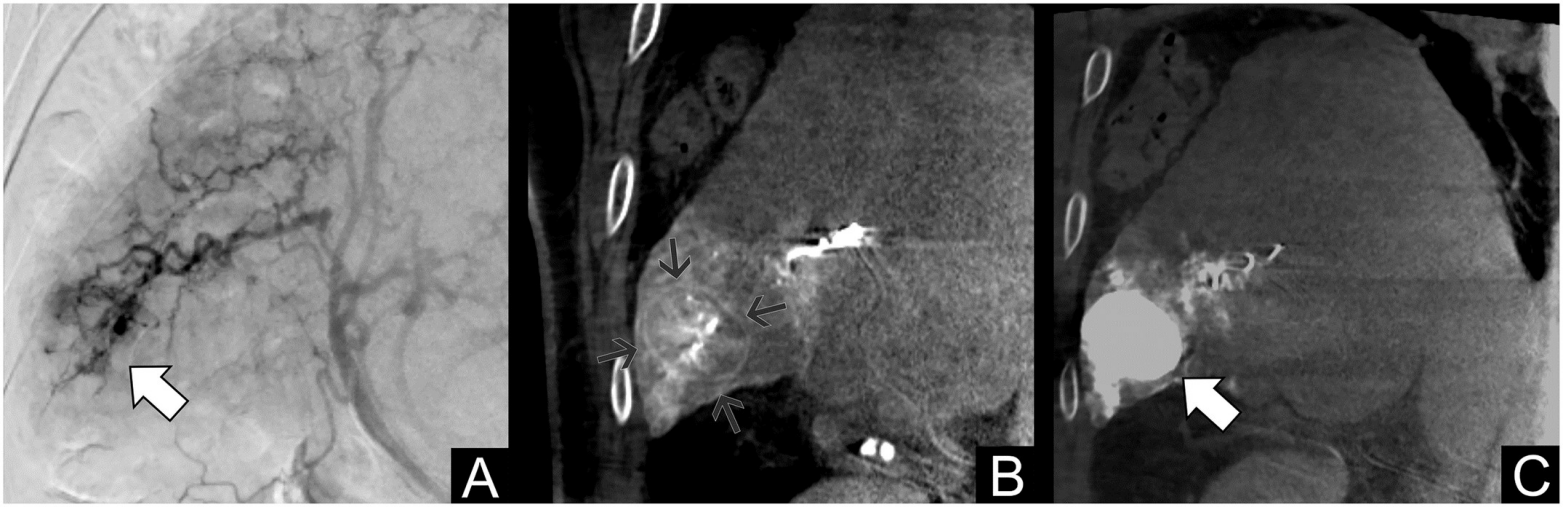
Patient #130, treated with TAE for multifocal HCC at 68 years (BCLC B, HCV+). A: Digital subtraction angiography of the right hepatic artery showing an HCC nodule in the sixth segment. B: Cone-Beam CT (coronal reconstruction) during contrast injection, highlighting the nodule capsule. C: Non-contrast final control after TAE showing marked contrast retention inside the lesion and complete arterial blood flow stoppage. The patient died during observation, with a survival of 21.1 months.

The goal of all TAE procedures is to achieve maximum ischemia in the tumor territory, sparing as much normal parenchyma as possible. Ideally, a catheter and a microcatheter are negotiated into small branches of the hepatic arterial tree, close to the tumor, and highly-ischemic microparticles (Embozene 40 or 100 μm, Boston Scientific, Marlborough, USA) are injected [11], to obtain tumor necrosis while preventing the development of collateral arterial flow [12]. The injection is continued until complete stasis was achieved and before retrograde reflux is seen (Fig 2).

#### Microparticles plus cyanoacrylate glue TAE (G-TAE)

The addition of cyanoacrylate glue to both TAE and TACE has been previously reported [13,14], with evidence of safety and increased complete response rates in one retrospective review. In our cohort, a 0.2 mL volume of a highly diluted (1:10) mixture of NBCA-MS (Glubran 2, GEM, Viareggio, Italy) with ethiodized oil (Lipiodol, Guerbet, Villepinte, France) was deployed for permanent sealing. [15–17].

**Fig 3:**
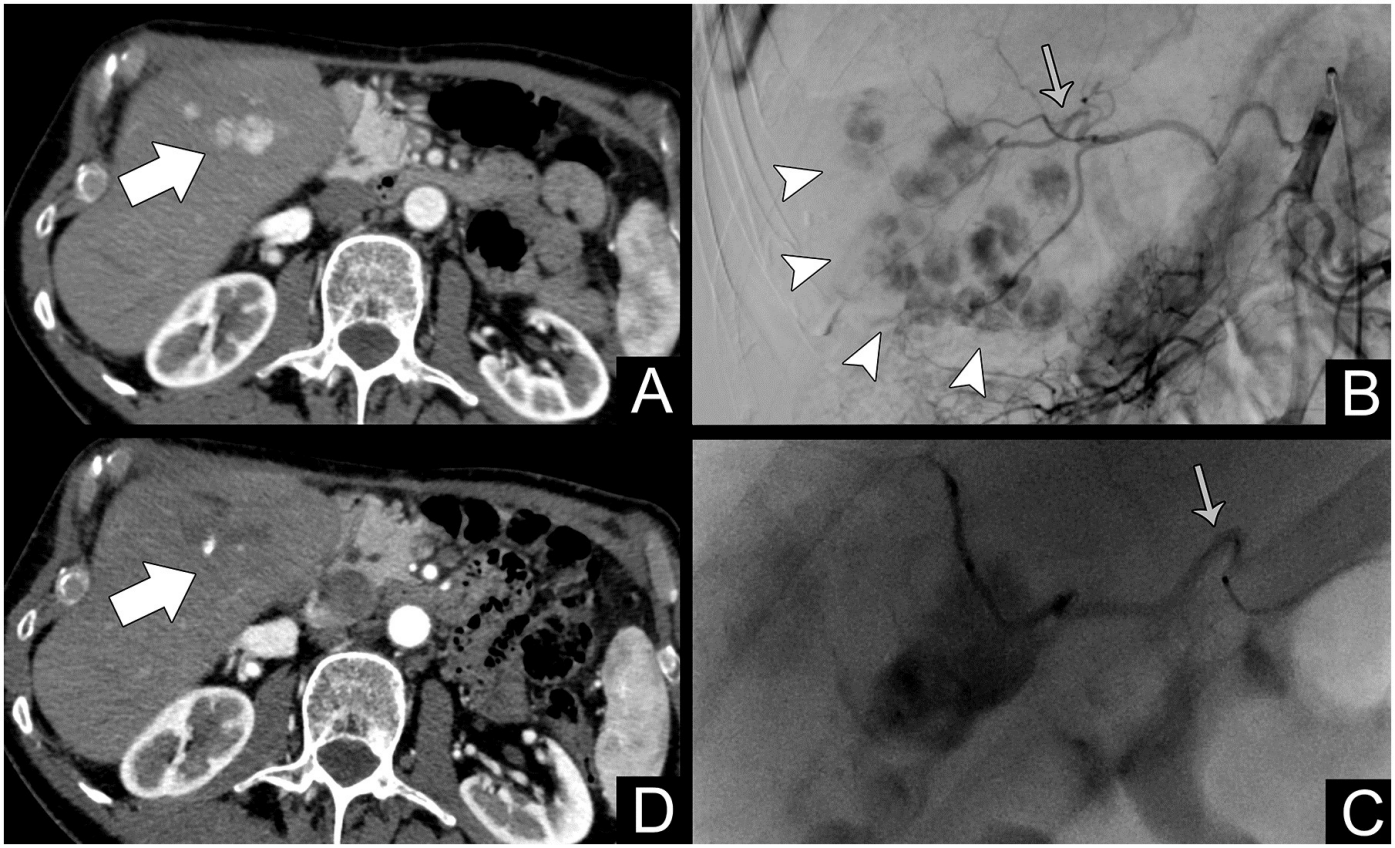
Patient #267, treated for multifocal HCC at 72 years (BCLC B). A: Contrast-enhanced CT arterial phase showing a multifocal hypervascular HCC in the fifth segment (white arrow); B: Selective angiography of the right hepatic artery showing the extent of the disease (white arrowheads). The gray arrow points to the main feeding vessel; C: Superselective catheterization (gray arrow) and embolization of the main feeding vessel (with *G*-TAE); D: One-month follow-up showing complete devascularization with a small hyperdense spot representing residual Lipiodol (white arrow). The patient was still alive at the end of the study with a survival of 17.4 months.

G-TAE was considered when a single tumor feeder was evident at angiography and successfully negotiated with the microcatheter. The choice of performing a G-TAE rested with the operator, who deemed feasibility and risks of non-target embolization case by case. Fig 3 shows the relevant imaging of a G-TAE.

#### Lipiodol TAE (L-TAE)

L-TAE is the injection of Lipiodol from a more proximal stance than P-TAE when the nodule feeding arteries are multiple, too contorted or too small to be catheterized super-selectively. No microparticles are used in L-TAE; the embolizing agent is instead ethiodized oil which induces sinusoidal congestion, determining hypoxia and necrotic areas surrounded by inflammatory cells. The ischemic effect is transient [18] and is thus considered a safer option when embolization of healthy parenchyma cannot be avoided.

### Statistical analysis

We used Stata 13 (StataCorp LP, Texas, USA) for all calculations. Survival analysis was performed by (LE) who has five years of experience in medical statistics. Cox regression was used for multivariate analyses. Death was considered the main event and was ascertained using the local, regional registry of Lombardia (Italy).

Predictors of survival were assessed using univariate analyses and log-rank tests for categorical variables. Kaplan-Meier curves were also depicted. We also analyzed a subgroup of patients who received concurrent PT to assess whether the time of such intervention (before or during the observation period) was a predictor of survival.

## Results

We retrieved 270 patients who underwent 646 liver embolizations for unresectable HCC from our records. Forty patients (15%) were excluded for having received a total of 11 TACE and 37 TARE. Finally, we included 230 patients (M = 181, F = 49, mean age = 73, SD = 8.1) who underwent 543 TAEs: 228 G-TAE (42%), 227 P-TAE (42%) and 88 L-TAE (16%). Table 1 summarizes the characteristics of our cohort.

**Table 1:** Cohort characteristics.

Sex	M	F	Overall	
	181	49	230	
**mean age (SD)**	72 (8.1)	75 (7.7)	73,5	
**median OS (months)**	27.5	30.5	28	
	**+**	**-**		
**HBV**	24	206		
**HCV**	127	103		
**Alcoholism**	45	2		
	*yes*	*no*		
**Percutaneous Treatment**	89	141		
**Previous surgery**	49	181		
**Secondary tumor**	33	197		
**Liver transplant**	2	228		
	**-**	**A**	**B**	
**Child-Pugh**	9	183	38	
	**-**	**0**	**A**	**B**
**BCLC**	1	23	95	111
**P-TAE**	227	42%		
**G-TAE**	228	56%		
**L-TAE**	88	16%		
**Total TT**	543			
**Avg TT per patient**	2,4			

OS is in months; TT = treatment;

HCV positive patients were 127 (55%), whereas HBV positive patients were 24 (10%). Among patients without HCV or HBV infection, 47 (20%, two females) had a history of alcoholism.

Previous liver resection was recorded in 49 (21%) patients. Two patients (1%) had received prior liver transplantation. Eighty-nine (39%) patients had received PT, 50 (22%) before the observation, and 38 (16%) during the time interval considered for this study as a result of down-staging. In the first ones, TAE was performed after PT consequently to a progression of the disease; in the latter cases, PT was performed in same nodules treated with TAE showing only partial response to embolization at first radiological control, meeting the BCLC criteria and after a multidisciplinary evaluation. Ablations included RF (58%), PEI (17%), MW (12%), and combinations of them (11%), as shown in Table 2.

**Table 2:** Types of ablation before and during the observation.

Percutaneous Ablation	Before observation	During observation	Total
RF	37	14	51
PEI	6	9	15
MW	4	9	11
RF + PEI	2	3	5
MW + RF + PEI	1	0	1
RF + MW	1	2	3
MW + PEI	0	1	1
Total	51	38	89

RF = radiofrequency; PEI = percutaneous ethanol injection; MW = microwave

Thirty-three (14%) patients had concurrent cancer, or this was discovered during follow-up.

HCV positive patients were 127 (55%), whereas HBV positive patients were 24 (10%). Among patients without HCV or HBV infection, 47 (20%, two females) had a history of alcoholism.

Child A was observed in 183 (80%), Child B in 38 (16%), in 9 patients, we were unable to retrieve sufficient data to calculate the score. Regarding BCLC classification, 23 (8%) were stage 0, 95 (41%) patients were stage A, 111 (48%) B, and 1 patient had no available data. All stage 0 patients had concurrent cancer withholding liver surgery. All stage A patients were directed to TAE for being unfit for surgery or for having an early disease recurrence after surgery.

Observation time ranged from 0.1 to 86.7 months (mean = 34.7, SD = 22.7); 121 (50%) patients died during this period, 8 (3%) were lost to follow-up and their survival time was retrieved by the last available check-up, 106 (46%) were still alive at the end of the observation.

OS rates at 1-, 2-, 3-, 4- and 5-year were of 84,8%, 58,7%, 38,3%, 28,3%, and 18,7%, as shown in Table 3.

**Table 3:** Survival rates at different time points grouped by BCLC stage and history of percutaneous treatment.

SURVIVAL RATES																
BCLC	baseline		1 year		2 yrs		3yrs		4 yrs		5 yrs		6 yrs		7 yrs	
-	1	0,4%	1	0,4%	1	0,4%	1	0,4%	1	0,4%	1	0,4%	1	0,4%	1	0,4%
0	23	10,0%	19	8,3%	17	7,4%	12	5,2%	9	3,9%	7	3,0%	2	0,9%	0	0,0%
A	95	41,3%	83	36,1%	60	26,1%	39	17,0%	28	12,2%	18	7,8%	6	2,6%	1	0,4%
B	111	48,3%	92	40,0%	57	24,8%	36	15,7%	27	11,7%	17	7,4%	6	2,6%	1	0,4%
no PT	141	61,3%	112	48,7%	72	31,3%	44	19,1%	31	13,5%	19	8,3%	7	3,0%	2	0,9%
PT	89	38,7%	83	36,1%	63	27,4%	44	19,1%	34	14,8%	24	10,4%	8	3,5%	1	0,4%
**TOTAL**	230	100%	195	84,8%	135	58,7%	88	38,3%	65	28,3%	43	18,7%	15	6,5%	3	1,3%

BCLC = Barcelona Clinic Liver Cancer stage, PT = patients who received a Percutaneous Treatment. The percentages are referred to the whole cohort (n = 230)

Regarding prediction of OS, univariate analyses resulted in inclusion of the following continuous variables in the final model (p = 0.2 cut-off for inclusion): total treatments received (p = 0.011, 95% C.I. -0.280 | 0.035), G-TAE treatments (p = 0.113, 95% C.I -0.310 | 0.033), P-TAE treatments (p = 0.124, 95% C.I -0.293 | 0.035), age upon entry in the study (0.067, 95% C.I -0.002 | 0.048). The log-rank tests for categorical variables (p = 0.2 cut-off) resulted in inclusion of having received a PT (p = 0.013). Results are shown in Table 4.

**Table 4:** Univariate statistical analyses.

Variable	Occurrence	p-value
Percutaneous Treatment	42	0.01
Sex	F = 26 M = 95	0.4
Age upon entry	mean 73.5	0.07
Child-Pugh score	A = 93 B = 23	0.3
BCLC stage	Stage 0 = 10 A = 55 B = 56	0.5
Previous surgery	23	0.2
Liver Transplant	1	0.5
Concurrent tumor	18	0.6
HCV+	69	0.9
HBV+	8	0.2
Total TAE treatments	543	0.01

Log-rank tests for categorical variables, Cox univariate analysis for continuous variables.

**Fig 4:**
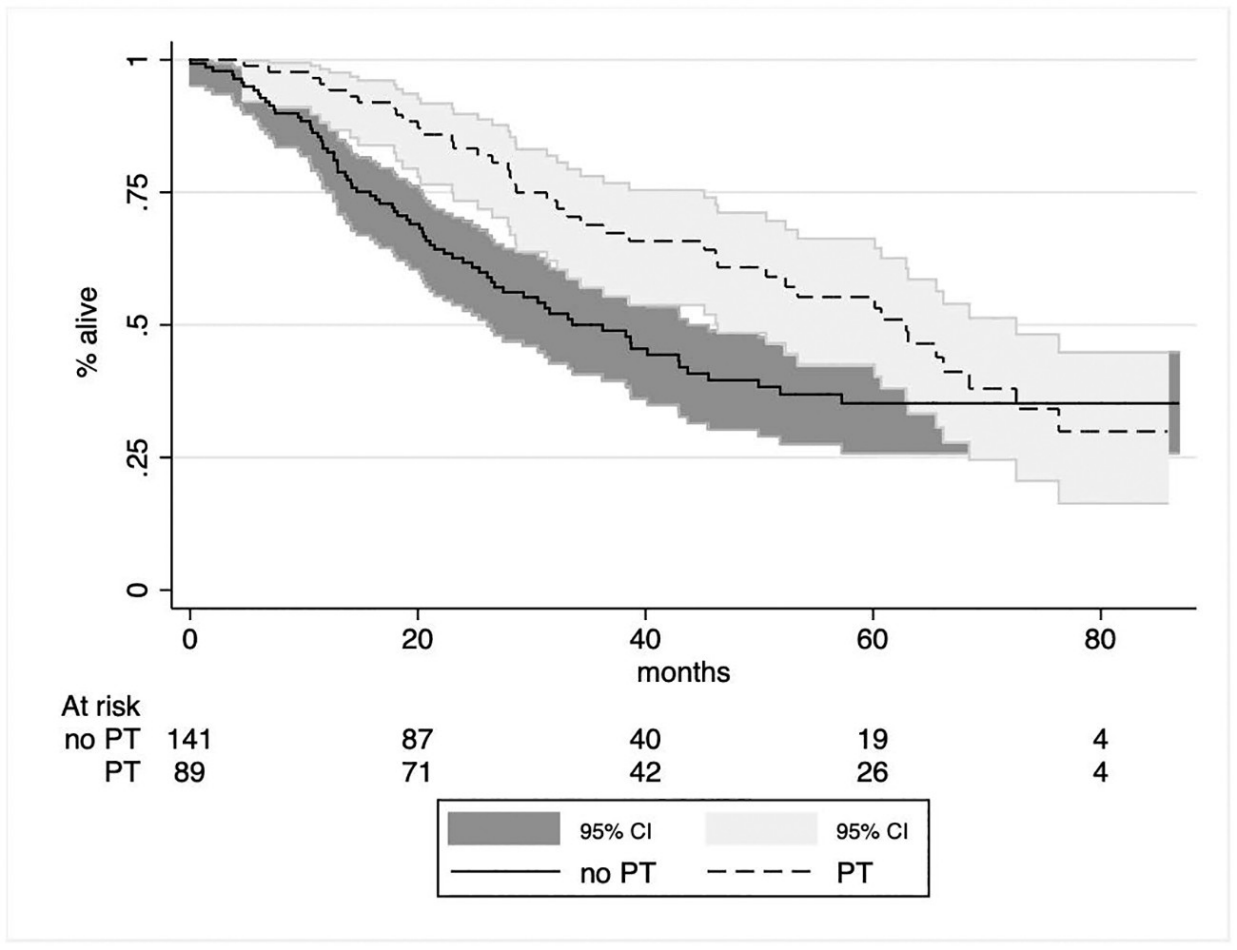
Kaplan-Meier survival graph grouped by percutaneous treatment: X-axis = survival in months; Y-axis = survival estimate.

We then performed a Cox regression test (p<0.05 was considered significant) including potential predictors (PT, previous surgery, HBV+, total treatments, age at first treatment). Two predictors of survival were identified: the total number of embolizations received (p = 0.04, HR = 0.779, 95% C.I 0.778 | 0.994) and having undergone a PT (Fig 4, p = 0.021, HR = 0.633, 95% C.I 0.430 | 0.933), whereas the other variables were non-significant.

## Discussion

In this cohort of patients with unresectable HCC treated only with TAE, we reported survival rates of 85% at one year and 59% at two years. These results align with the highest OS rates reported in a meta-analysis for TACE, with 82% and 63% for 1-year and 2-years survival, respectively [19].

We analyzed eight TAE papers [20–27], dated from 1991 to 2014, and reported 1-year OS from 42% to 86% and 2-yrs OS from 25% to 51%. Notably, only two groups adopted microparticle embolization similar to our P-TAE [23,27], whereas the rest employed L-TAE and none used cyanoacrylates. This highlights a lack of data regarding cohorts treated with small calibrated microparticles (40–100μm) and cyanoacrylates, which are especially needed since recent papers have suggested their better performance in terms of local tumor control rates [11,13,28,29]. The use of small-sized particles has in fact already improved OS for TACE procedures, as reported recently by Prajapati et al. who noted an OS of 15.1 for patients treated 300–500μm beads versus 11.1 months for the 500–700μm group [30].

TACE is the most adopted technique for advanced-stage HCC, despite being more expensive and worse tolerated than TAE [2,31]. However, the added value of chemotherapy is controversial. Two systematic reviews support the non-superiority of TACE over TAE [2,32] and a recent network meta-analysis [33] concluded that TACE, DEB-TACE, TARE and adjunctive systemic agents (combined with TACE or DEB-TACE) did not improve OS compared to bland TAE.

Distal embolization and ischemia challenge chemotherapy as the main factor for tumor control for HCC. Therefore, TAE with Lipiodol, which doesn’t have the same ischemic effect as particle embolization, should be used only when particle embolization is unfeasible (such as in inaccessible HCCs or advanced multifocal disease), as in the present study where L-TAE was adopted only in 16% of interventions.

Growing evidence, mainly based on data regarding TACE, supports that combining endovascular and percutaneous treatments increases efficacy [34]. This trend is also confirmed in our TAE study, where PT predicted a better OS increasing the chance of survival by 37%. PT is normally proposed for BCLC 0-A stage HCCs when less than three nodules under 3 cm in size are present [35], but an efficacious TAE may be used to downstage the disease and allow for PT, as occurred in 16% of our patients.

There are limitations to this retrospective survival analysis. First, we did not compare the different techniques of treatment (e.g., P-TAE vs. G-TAE) since patients were not allocated “a priori” to a single treatment group and the choice among treatments may also have been influenced by the operators’ experience. Second, there was no evaluation of the time-to-progression rates between treatments, which may have highlighted differences between techniques. Last, we were unable to identify the cause of death, particularly relevant to patients who had a concurrent tumor (13%).

**Fig 5:**
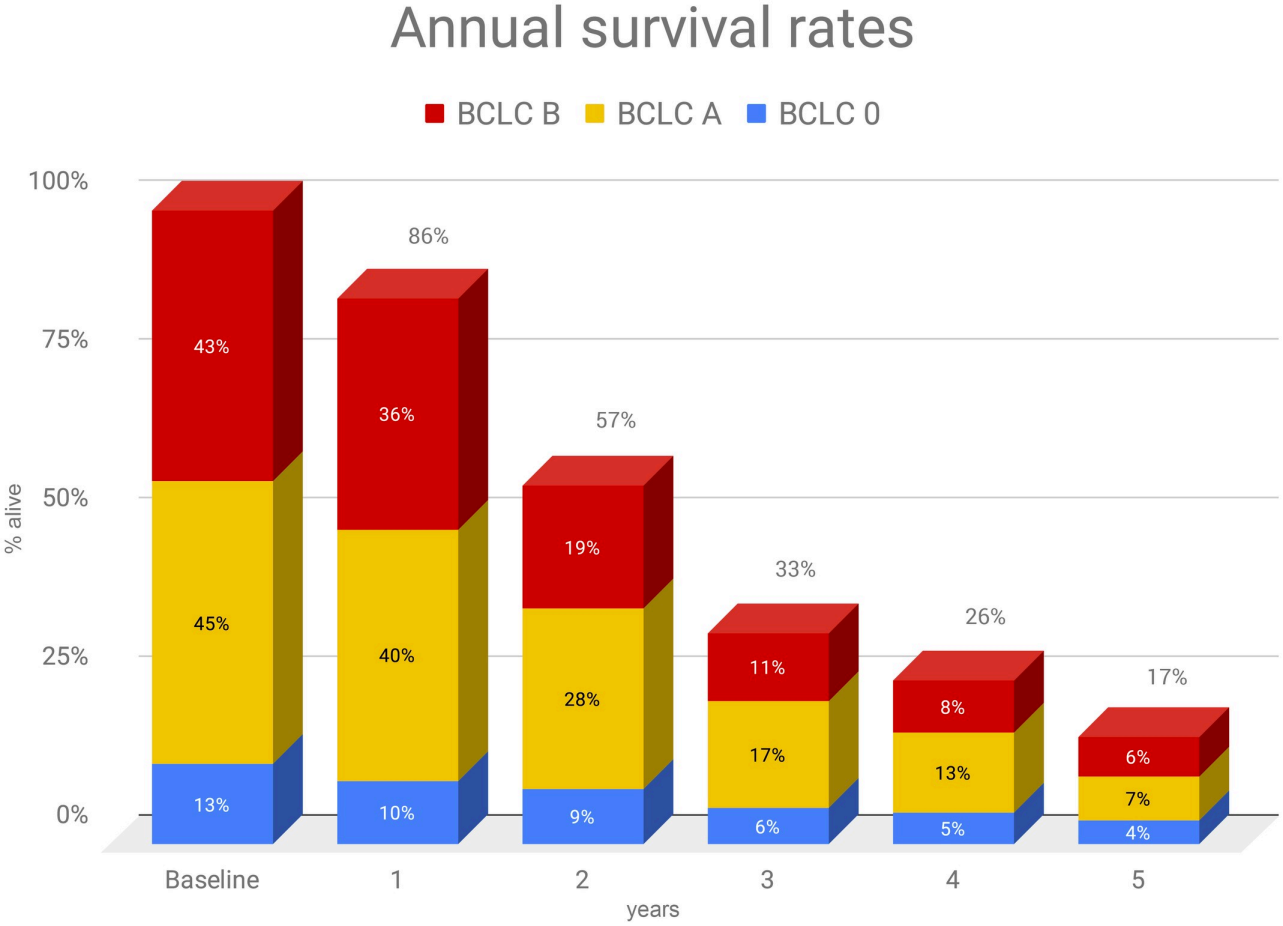
Histogram of annual survival stratified for the BCLC stage of patients. X-axis = survival in years; Y-axis = survival estimate.

We reported non-inferior survival rates compared to other published studies adopting TACE. Importantly, this evidence is by no means derived by a direct comparison of TAE vs. TACE in the same group. However, many studies as such are available and a recent meta-analysis [2] has already proved such non-inferiority with a high level of evidence. On the contrary, studies reporting long-term survival rates of bland embolization alone are uncommon. The strengths of this paper are in facts the large cohort and the long-term follow-up of patients treated exclusively with TAE. Notably, the OS rates were not significantly modified by disease stage or underlying hepatopathy (Fig 5), suggesting an opportunity for treatment also in advanced HCCs.

Our results add to the body of literature currently questioning the need for adding chemotherapeutic drugs to endovascular treatments for HCC. We evidenced the survival rates achievable using TAE alone, particularly the importance of adopting small-sized microparticles and of adding NBCA-MS when deemed safe, in order to improve survival rates in these patients.

## Supporting information

S1 Table Patients’ database. A STATA format file, which contains characteristics of the cohort, survival rates, types of intervention performed and all data used for statistics.
(DTA)
